# Supplementary material for: Nonhuman Primates Satisfy Utility Maximization in Compliance with the Continuity Axiom of Expected Utility Theory
Source: J Neurosci. 2021 Mar 31;41(13):2964–79. doi: 10.1523/JNEUROSCI.0955-20.2020 (PMC8018892; doi:10.1523/JNEUROSCI.0955-20.2020)
Supplement: Figure 4-1 — Comparison of ICs' fitting models. Mean squared error (MSE) resulting from fitting IPs to three different models. Bold face indicates the lowest MSE value for each animal. Download Figure 4-1, DOCX file. [file ns-JN-RM-0955-20-s01.docx]

Table 3-1

| *Model*  Monkey | *Linear* | *Hyperbolic* | *Power* |
| --- | --- | --- | --- |
| A | 1.1⋅10^-2^ | 9.9⋅10^-5^ | **9.6⋅10^-5^** |
| B | 3.4⋅10^-2^ | 2.2⋅10^-4^ | **1.7⋅10^-4^** |
