# Supplementary material for: Nonhuman Primates Satisfy Utility Maximization in Compliance with the Continuity Axiom of Expected Utility Theory
Source: J Neurosci. 2021 Mar 31;41(13):2964–79. doi: 10.1523/JNEUROSCI.0955-20.2020 (PMC8018892; doi:10.1523/JNEUROSCI.0955-20.2020)
Supplement: Figure 6-1 — Comparison of economic models. Each row of values is a comparison across models using one regression model accuracy metric (averaged across all tests and sessions), with bold face indicating the best fitting model according to that metric (gray font for the worst fitting one). EV corresponds to the EU model assuming a linear utility function. In the PW model the gamble value was computed as V = U(m)⋅ w(p) (valid, as defined in Prospect Theory, for all gambles with one non-zero outcome), with w(p) being the PW function (2-parameter Prelec function). In the additive model, V=wm⋅U(m)+wp⋅p. The square root of the MSE represents the average distance between model and IP in probability units. Var represents the variance in the differences of modeled versus measured preferences (the proportion of AC vs B choices across all continuity tests). Download Figure 6-1, DOCX file. [file ns-JN-RM-0955-20-s02.docx]

Figure 6-1

|  | *Model*  *Metric* | *EV*  *U: linear*  *w: linear* | *EU*  *U: power*  *w: linear* | *EU*  *U: Prelec*  *w: linear* | *PW*  *U: power*  *w: Prelec* | *Mean-variance* | *Additive 1*  *U: Prelec*  *w: linear* | *Additive 2*  *U: power*  *w: Prelec* |
| --- | --- | --- | --- | --- | --- | --- | --- | --- |
| Monkey A | $\sqrt{MSE}$  *BIC*  *AIC*  *Var* | 0.108  348  344  0.154 | 0.046  296  288  0.122 | 0.028  **293**  281  0.117 | **0.024**  294  **275**  **0.113** | 0.066  308  300  0.129 | 0.091  347  331  0.142 | 0.126  320  301  0.125 |
| Monkey B | $\sqrt{MSE}$  *BIC*  *AIC*  *Var* | 0.274  379  376  0.225 | 0.093  324  317  0.176 | **0.052**  316  305  0.165 | 0.062  **310**  **292**  **0.155** | 0.193  342  335  0.197 | 0.212  333  319  0.174 | 0.084  330  312  0.168 |
